# Hepatitis B Virus (HBV) Variants in Untreated and Tenofovir Treated Chronic Hepatitis B (CHB) Patients during Pregnancy and Post-Partum Follow-Up

**DOI:** 10.1371/journal.pone.0140070

**Published:** 2015-10-16

**Authors:** Boris Virine, Carla Osiowy, Shan Gao, Tong Wang, Eliana Castillo, Steven R. Martin, Samuel S. Lee, Kimberley Simmonds, Guido van Marle, Carla S. Coffin

**Affiliations:** 1 Liver Unit, Division of Gastroenterology and Hepatology, Cumming School of Medicine, University of Calgary, Calgary, AB, Canada; 2 Department of Microbiology, Immunology and Infectious Diseases, Cumming School of Medicine, 3280 Hospital Drive NW, University of Calgary, Calgary, AB, Canada; 3 Bloodborne Pathogens and Hepatitis Laboratory of the National Microbiology Laboratory, Winnipeg, MB, Canada; 4 Artifical Liver Centre, Beijing YouAn Hospital, Capital Medical University, Beijing, China; 5 Maternal Disorders in Pregnancy, Section of Internal Medicine, Department of Medicine, Cumming School of Medicine, University of Calgary, Calgary, AB, Canada; 6 Department of Pediatrics, Alberta Children’s Hospital, Cumming School of Medicine, University of Calgary, Calgary, AB, Canada; 7 Alberta Health, Government of Alberta, Edmonton, AB, Canada; University of Cincinnati College of Medicine, UNITED STATES

## Abstract

**Background:**

Chronic hepatitis B (CHB) is a dynamic disease that may be affected by immune changes in pregnancy. Guidelines suggest consideration of nucleos/tide analogs (NA), i.e., tenofovir, (TDF) in highly viremic mothers to reduce vertical transmission risk. HBV variability affects CHB outcome, but little is known about HBV genetic changes in pregnancy due to immune or NA selection.

**Objectives:**

To evaluate HBV diversity in NA treated or untreated pregnant vs. post-partum CHB carriers.

**Study Design:**

In plasma collected from 21 mothers (7 matching pre/post-partum), HBV serological tests, genotype and viral load were assayed. The HBV pre-surface (S) /S overlapping polymerase (P) (N = 20), pre-core (C) /C (N = 11) and/or full genome PCR amplicons (N = 3) underwent clonal sequence analysis.

**Results:**

The median age was 31 y, 71% Asian, 68% genotype B or C, 33% HBV eAg+, 5 received TDF (median HBV DNA 8.5 log IU/ml). In untreated mothers, median antepartum vs. post-partum ALT was 21 vs. 24 U/L and HBV DNA was 2.7 vs. 2.4 log(10) IU/ml. ALT and/or HBV DNA flares occurred during pregnant and/or post-partum period in 47% (10/21). Clonal sequencing antepartum showed the presence of minor “a determinant” and/or vaccine escape mutants (VEM) but drug resistant variants were infrequent. Analysis of pregnant vs. post-partum samples showed different HBV variants and viral diversity.

**Conclusions:**

Differences in immune and/or by NA selective pressures during pregnancy may affect HBV evolution during pregnancy. The presence of minor VEM warrant infant follow-up.

## Introduction

Chronic hepatitis B virus (HBV) infection (CHB) remains a global health problem, with ~240 million people infected worldwide [1], who remain at risk of end-stage liver disease and hepatocellular carcinoma (HCC) [2–4]. In HBV endemic areas, vertical or mother to child transmission (MTCT) is an important mode of infection [1]. The administration of HBV immunoprophylaxis consisting of vaccination with recombinant HBV surface (S) protein, along with hepatitis B immune globin (HBIG) at birth has significantly reduced the global burden of CHB [5]. However, immunoprophylaxis failure continues to occur and is linked to emergence of HBV “vaccine escape mutants” (VEM), as well as maternal HBV e antigen (HBeAg) positivity and high maternal viral loads (>6 log-10 virus copies/ml) [6–9]. Thus, expert clinical guidelines advocate the use of HBV reverse transcriptase (RT) / polymerase (P) inhibitors (i.e., nucleoside/nucleotide analogs, NA) in highly viremic mothers to reduce the risk of MTCT and immunoprophylaxis failure [10–12].

HBV replicates via an error-prone RT, leading to significant viral diversity [13, 14]. Due to overlapping reading frames, changes in the HBV S / envelope encoding region may result in changes in the HBV P and vice versa [15]. The “a” determinant of the HBV S region is a common immunodominant region shared by different genotypes of the HBV, spanning amino acids (aa) 124 to 149, and forms a conformational antigenic loop, stabilized by cysteine-disulfide bonds [16]. Mutations or variants within the S/P as well as the immunodominant “a determinant” region have been implicated in the development of immune escape and antiviral resistance [6]. Other studies have also demonstrated that although the major antigenic binding epitopes of HBsAg may not be affected, distant mutations can modulate the a-determinant by influencing epitope conformation. Mutations in the HBV pre-S1 and pre-S2 region due to increased host immune pressure have also been associated with liver disease progression and increased risk of HCC [17, 18].

CHB is a heterogeneous dynamic the outcome is determined by host antiviral immune response which may be further modulated by host immune changes that occur in pregnancy and the post-partum period, as well as the use of antiviral therapy [19, 20]. The HBV is a non-cytopathic virus and acute hepatic flares during immune activation occur as a result of a robust HBV-specific T cell response and can lead to liver injury [21]. During pregnancy a number of physiological changes can occur that may affect the course of CHB. The maternal immune system does not “respond” to the presence of allogeneic (i.e., “non-self”) foetal antigens in a complex physiological adaptation to allow successful fetal development [22]. These changes might allow greater viral replication, followed by reconstitution of the immune system post-partum and reactivation flares. In clinical studies, overall variable outcomes have been reported during pregnancy. In our recent study of 161 treated and untreated patients, we observed frequent presence of mild-moderate ALT flares post-partum [23]. Ayres *et*. *al*. [24] determined by next generation sequencing analysis that even short-duration lamivudine (LMV) therapy in pregnancy significantly increased viral quasispecies diversity. Despite the importance of HBV diversity for HBV pathogenesis, there is little data on HBV variants during pregnancy especially with the use of more potent NA therapy such as TDF. In the current study we aim to evaluate the genetic diversity of the HBV and to determine the presence of variants associated with immune escape and drug resistance in TDF- treated and untreated pregnant and post-partum CHB carriers.

## Materials and Methods

### Patients and samples

Ethics approval for this study was granted by the University of Calgary Conjoint Health Research Review Board in accordance with the Declaration of Helsinki (Ethics ID #16636 and #25084). All subjects provided written informed consent to participate. In total, 21 HBV mono-infected pregnant patients (with 22 healthy pregnancies) participated in this study. All patients were referred for assessment in the second trimester and followed until ~ 6–12 months post-partum. CHB infection was diagnosed according to the Government of Alberta (Alberta Health) prenatal screening program by the presence of hepatitis B surface antigen (HBsAg) in serum. The clinical and demographic data recorded included age, sex, co-morbid liver disease and antiviral therapy. Laboratory information included HBV alanine aminotransferase (ALT) levels. HBV serologic markers included HBsAg, HBV e antigen (HBeAg), HBV surface antibody (anti-HBs), and HBe antibody (anti-HBe), were evaluated by commercial chemiluminescent microparticle immunoassays (Abbott ARCHITECT, Abbott Laboratories, Mississauga, ON, Canada). The HBV DNA levels in serum were tested by real time PCR (detection limit <10 IU mL-1 or ∼50 copies mL-1, Abbott Architect, Abbott Laboratories, Mississauga, ON, Canada). HBV genotype was determined by line probe assay (INNO-LiPA, Innogenetics N.V., Ghent, Belgium). CHB flare was defined as >2-fold increase in ALT, and/or >2-log10 increase in HBV DNA from baseline. Liver stiffness measurement by transient elastography (TE, FibroScan^®^, Echosens, France) was done post-partum to assess liver fibrosis.

### Clonal Sequence Analysis of Overlapping HBV Polymerase/Surface Gene and Pre-Core / Core Gene in Pregnant/Post-partum CHB carriers

In total we completed clonal sequencing of the pre S/S and/or pre C/C region in 21 patients (22 healthy pregnancies), including 7 cases with matched samples collected in the pregnant or post-partum period (ID# 160, 176, 196, 215, 223, 226, and 233). In consented patients, plasma was isolated from whole blood and stored at -80°C. Total DNA were isolated from ~200–1000 mL-1 of plasma using commercial nucleic acid extraction kits (E.Z.N.A.^®^ HP Viral RNA/DNA Kit R6873-00, Omega, Norcross, GA), or by classical phenol-chloroform/ethanol precipitation method. HBV genomes were amplified using HBV Pre-S/S/overlapping P, pre-C/C or full genome primers via direct and, if required, two-round nested PCR, using previously published PCR primers and conditions [25, 26]. The nucleotide location and fragment size of the pre-S/S primers were as follows: 1st round: forward 2820–2840, reverse1196-1175, size of fragment 1597 bp; 2nd round, forward 2816–2839, reverse 899–878, size of fragment 1298 bp. The Pre-C/C primers were as follows: 1st round: forward1606-1626, reverse 2366–2385, size of fragment 780 bp; 2nd round forward1825-1843, reverse 2366–2385, size of fragment 462 bp. PCR products were agarose-gel purified (GenElute^™^ Gel Extraction Kit, Sigma-Aldrich, Oakville, ON, Canada) and cloned using the TOPO^®^ TA Cloning^®^ Kit (Life Technologies, Carlsbad, CA). Following plasmid isolation using the GenElute^™^ Plasmid Miniprep Kit (Sigma-Aldrich Canada, Oakville, ON, Canada), ~10 plasmid inserts/sample were sequenced with universal primers and 3730 XL sequencing system (Applied Biosystems, Foster City, CA, USA). The cloned sequences were sequenced bidirectionally, and variant nucleotides confirmed by visual analysis of chromatographs.

### Data Analysis

Clonal sequences were translated for S and P, and C protein and aligned with sequences of the same genotype (Genbank Accession# KT723028-KT723432; KT820058-KT820067). Genbank reference sequences were used for alignment. Genotype was determined with the NCBI genotyping tool [27]. Molecular and evolutionary sequence analysis was conducted using MEGA software V 6.0 [28]. Phylogenetic analysis was conducted using the Kimura-2-parameter substitution method and 1000 bootstrap replicates to construct a neighbour joining tree. Distance calculations were used to assess the relative evolutionary divergence between samples. To evaluate the nature of the selection pressures on the viral sequences a Z-test of selection was conducted to test the hypotheses of neutral (synonymous = non synonymous mutations), positive (synonymous < non synonymous mutations) and purifying (synonymous >non synonymous mutations) selection. The analysis was conducted using the Nei-Gojobori method [29], and the variance of the difference was calculated using 2000 bootstrap replicates. Statistical analysis was done using STATA. An analysis of variability (ANOVA) with appropriate post-hoc test was used for the comparison of continuous data and the γ ^2^ or Fischer’s exact test was used for the comparison of categorical data. Two-tailed *P* values <0.05 were considered significant.

## Results

### Summary of Maternal and Infant Clinical and Virological Data

There were 21 pregnant CHB carriers (with 22 infants, 1 mother had 2 pregnancies) enrolled (Table 1) with median age of 31 years (range 21–37), 15/21 (71%) Asian, 13/20 (65%) were genotype B or C determined by line probe assay, 33% (7/21) HBeAg positive, and median baseline vs. post-partum ALT was 21 U/L (range 6–47) vs. 24 U/L (range 7–64). Five patients were treated with TDF after 28 weeks of gestation to reduce viral load prior to delivery (median HBV DNA 8.5 log (10) IU/ml, ID# 150, 223, 233, 239 and 243); 1 mother with HBV DNA >8.0 log (10) IU/ml declined therapy (ID# 205). In untreated patients, median baseline vs. postpartum HBV DNA was 2.7 vs. 2.4 log (10) IU/ml. In 10/21 (47%) of cases (ID #133, 146, 150, 160, 161, 172, 196, 215, 223, and 239) ALT and/or HBV DNA flares were observed in pregnancy and/or post-partum. All patients had normal post-partum liver stiffness measurement (LSM) by transient elastography (median LSM 4.7 kpa, range 2.8–6.1). According to Alberta Health centralized reporting data, 68% of infants (15/22) have completed HBIG and the 3-dose HBV vaccine series, and have confirmed protective immunity (i.e., HBsAg negative, anti-HBs titres > 10 IU/ml) at the time of this reporting. However, in 31% (7/22 infants) data is unknown, or infant immunoprophylaxis and testing is incomplete (Table 1). There were no reported cases of immunoprophylaxis failure.

**Table 1:** Clinical Data in 21 Pregnant and/or Post-partum CHB Patients with 22 Infants who Received HBV Immunoprophylaxis.

ID N = 21	Age/Ethnic	Geno-type LiPa^a^	HBV DNA log (10) IU/mL pre/ post^b^	HBe/Anti-HBe	ALT ^c^ (U/L), pre/ post-partum	TE kpa^d^	Delivery Mode^e^, Antiviral	Infant HBIG+3-dose Vaccine/ Follow-up HBV Serology^f^
128	36/Asian	B	<1.7 / 1.9	Neg / Pos	15 / 17	3.0	SVD, No	Completed/ HBV Protected
133	31/Asian	B	2.3 / 2.2	Neg / Pos	10 / 24	Unk	Emerg C/S, No	Completed/ HBV Protected
139	31/ Caucas	A	Unkn / < 1.3	Neg / Pos	23 / 19	3.2	SVD, No	HBIG + 1 dose/ Non-compliant
146	27/Asian	B	4.5 / 3.4	Neg / Pos	13 / 39	3.0	Elective C/S, No	Completed/ HBV Protected
150	21/ S. Asian	D	8.2 / Unkn	Pos / Neg	47 / 23	Unk	Vacuum-assist VD, Tenofovir	Completed/ HBV Protected
160	25/Asian	B	5.6 / 7.0	Neg	32 / 64	5.3	SVD, No	Completed/ HBV Protected
161	35/Asian	C	2.8 / 3.3	Neg /Unkn	26 / 47	5.8	SVD, No	Not Given/ Pending
172	24/Asian	C	2.7 / 3.9	Neg/ Unkn	16 / 64	Unk	SVD, No	Not Given/ Pending
176 (2 preg)	29/Asian	B	Unkn / 3.0 /< 1	Neg /Pos	Unk/ 18 / 7	5.0	Emergent C/S, No	Completed/ HBV Protected (n = 2)
188	30/Africa	E	3.2 / Unkn	Neg/ Unkn	34 / 16	4.5	Vacuum assist VD, No	Completed/ HBV Protected
196	31/Cauca	D	2.1 / <1	Neg/ Unkn	12 / 29	6.1	SVD, No	HBIG+2 doses/ Lost Follow-up
205	37/Asian	B	8.0 / Unkn	Pos / Neg	27 / 25	Unk	Emergent C/S, No Declined	Completed / HBV Protected
215	27/African	E	1.8 /3.1	Neg/ Unkn	43 / 41	4.9	Emergent C/S, No	Completed / HBV Protected
217	23/Asian	A	2.6 / 2.1	Pos / Unkn	29 / 19	2.8	Vacuum assist VD, No	Completed / HBV Protected
223	33/Cauca	D	8.8 / 4.2	Pos / Neg	20 / 56	Unk	SVD, Tenofovir	Completed/ HBV Protected
226	36/Asian	B	5.6 /4.9	Neg/ Unkn	29 / 32	3.8	SVD, No	Completed/ HBV Protected
231	37/ Asian	B	1.7 /2.4	Neg/ Pos	32 / 13	3.7	SVD, No	Unknown
233	29/Asian	C	8.8 /8.2	Pos/ Unkn	19 / 33	3.8	Vacuum assisted VD, Tenofovir	Completed/ Pending
239	29/Asian	B	8.0 /4.2	Pos/ Neg	6 / 26	Unk	SVD, Tenofovir	Completed/ Pending
243	34/Asian	C	8.5 /3.4	Pos/ Unkn	15 / 16	3.2	C/S, Tenofovir	HBIG+3 doses/ Protected
260	32/African	Unk	3.9 /3.1	Neg/ Pos	11/ 7	Unk	Forceps, Vacuum, No	HBIG+3 dose/ Protected

Notes:

^a^LiPA, INNO-LiPA HBV genotyping assay (Innogenetics N.V., Ghent, Belgium);

^b^HBV DNA tested by commercial PCR (Abbott RealTime HBV assay M2000, sensitivity <10 IU/ml or ~50 copies/ml, Abbott Laboratories, Mississauga, Ontario, Canada) during pregnancy and post-partum;

^c^ Normal alanine aminotransferase (ALT) is <20 U/L in females, and ALT flare is ~2.0 fold increase from baseline.

^d^TE, transient elastography (FibroScan^®^, Echosens, France) for liver stiffness measurement;

^e^SVD, spontaneous vaginal delivery; C/S, Caesarean section; HBIG, hepatitis B immune globin.

^f^In pending cases the infant has not reached the appropriate age to receive the 3-dose vaccine series or undergo testing for HBsAg and anti-HBs titres.

### HBV Genotype Distribution is Consistent with Ethnicity and Pregnant Chronic Hepatitis B Carriers Frequently Carry Minor Variants at Residues Associated With Vaccine Escape but Drug Resistant Variants are Rare

The dominant genotype of each patient based on clonal sequence analysis corresponded with ethnicity and line probe genotyping results in most cases. Clonal sequence analysis revealed that 2 cases (ID# 133, and 231) had a potential mixed or recombinant genotype infection (Table 2). In most cases (61%, N = 13/21) sequence analysis of the HBV pre-S1/ pre-S2, and S encoding region showed variants in the “a-determinant” region sites known to affect antigenicity (i.e., aa 124, 126, 129, 131, 133, 137, 139, 146, and 147) with median 10.4–12.5% of clones affected in pre and/or post-partum sample tested (Table 2, S1 Table). In 4 cases (#161, 176, 231, and 233) variants at positions associated with classic VEM (i.e., G145A/R, P120S) was found, albeit at low frequency. Cases #161, 188 and 226 had mutations at aa 129, which are linked to altered anti-HBs binding. In cases #150, 176, 188, 233 and 243 other variants found included those associated with disulfide bridge disruption that may affect the “a determinant” conformation (i.e., aa137, 139, and 147). Notably, in 1 case (ID #176) a mutation at the N-glycosylation site (aa 146) was detected. Cases #176, 215 and 231 carried the pre-S2 region deletion, at a frequency ranging from 14–66% (1/7–4/6 clones). In the overlapping P, potential mutations at residues associated with drug resistance were identified in Case #160 (rtL80V), and Case #217 (rtV191A) and a premature stop codon was found in Case #176 (rtQ182stop). The rtR153Q mutant in the HBV P that is associated with changes in the overlapping S (i.e., G145R, VEM) was also detected in Case #176. This variant was recently reported in association with virological breakthrough and drug resistance to Tenofovir in a patient on sequential anti-HBV therapy with multi-site P/RT mutations [30]. (S1 Table, Table 2).

**Table 2:** HBV Genotype and Frequency of S (Immune Escape) and P (Drug Resistant) Variants During Pregnancy vs. Post-Partum in 21 CHB Carriers by Clonal Sequencing Analysis.

Age, Ethnic/ID (Date Collected)	% (N clones) Pregnant vs. Post-partum; Genotype (%, N)	% (N clones) Pregnant vs. Post-partum; S (immune escape and liver disease) ^b^	% (N clones) Pregnant vs. Post-partum; P (drug resistance) ^d^
36 Asian / 128 (2011-11-16) 128–2 (2012-11-07)	B (100, 2) (pregnant) vs. B (100, 1) post-partum	0 (0/2) (pregnant)	0 (0/2) (pregnant)
31 Asian / 133 (2011-12-21)	C (91.7, 11/12), C/D (8.3, 1/12) (pregnant)	41.7 (5/12) and 58.3 (7/12) (pregnant)	0 (0/12) (pregnant)
31 Caucasian / 139 (2012-01-18)	C (100, 12/12) (pregnant)	8.3 (1/12) and 0 (0/12) (pregnant)	0 (0/12) (pregnant)
27 Asian / 146 (2012-03-20)	B (100, 9/9) (pregnant)	0 (0/9) ^c^ (pregnant)	Unknown
21 S. Asian / 150 (2012-04-12)	D (100, 7/7) (pregnant)	12.5 (1/8) and 0 (0/8) (pregnant)	0 (0/8) (pregnant)
25 Asian^a^ 160 (2012-08-01) 160–2 (2013-03-20)	B (100, 11/11) (pregnant) vs. B (100, 14/14) (post-partum)	0 (0/11) (pregnant) vs. 0 (0/14) (post-partum) and 0 (0/11) (pregnant) vs. 0 (0/14) (post-partum)	9.1 (1/11) (pregnant) vs. 0 (0/14) (post-partum)
35/Asian 161 (2012-08-08)	C (100, 7/7) (pregnant)	28.6 (2/7) and 100 (7/7) (pregnant)	0 (0/7) (pregnant)
24/Asian 172 (2012-10-31)	C (100, 17/17) (pregnant)	0 (0/17) and 64.7 (11/17) (pregnant)	0 (0/17) (pregnant)
129 Asian^a^ 176–2 (2013-01-09) 176–3 (2014-02-11)	B (100, 11/11) (pregnant) vs. B (100, 6/6) (post-partum)	90.9 (10/11) (pregnant) vs. 100 (6/6) (post-partum) and 0 (0/11) (pregnant) vs. 0 (0/6) (post-partum)	9.1 (1/11) (pregnant) vs. 20 (1/6) (post-partum)
30 African 188 (2013-01-09)	E (100, 12/12) (pregnant)	58.3 (7/12) and 100 (12/12) (pregnant)	0 (0/12) (pregnant)
31 Caucasian 196 (2013-02-06) 196–2 (2013-04-26)	D (100, 3/3 (pregnant) vs. D (100, 2/2) (post-partum)	0 (0/2) (pregnant) vs. 0 (0/3) (post-partum) and 0 (0/2) (pregnant) vs. 0 (0/3) (post-partum)	0 (0/2) (pregnant) vs. 0 (0/3) (post-partum)
37 Asian 205 (2013-03-20)	B (100, 5/5) (pregnant)	0 (0/5) and 0 (0/5) (pregnant)	0 (0/5) (pregnant)
27/African 215 (2013-06-03) 215–2 (2013-11-13) ^a^	E (100, 6/6) (pregnant) vs. E (100, 13/13) post-partum	0 (0/6) and 100 (6/6) (pregnant)	0 (0/6) (pregnant)
23 S. Asian 217 (2013-06-12)	A (100, 13/13) (pregnant)	15.4 (2/13) and 21.1 (3/13) (pregnant)	7.7 (1/13) (pregnant)
33 Caucasian 223 (2013-07-12)	D (100, 6/6) (pregnant)	0 (0/6) and 0 (0/6) (pregnant)	0 (0/6) (pregnant)
36 Asian^a^ 226 (2013-07-24) 226–2 (2013-11-19)	B (100, 8/8) (pregnant) vs. B (100, 10/10) (post-partum)	0 (0/8) (pregnant) vs. 20.0 (2/10) (post-partum) and 25.0 (2/8) (pregnant) vs. 40.0 (4/10) (post-partum)	0 (0/8) vs. 0 (0/10)
37 Asian 231 (2013-09-04)	B (100, 4/7), C (3/7) (pregnant)	85.7 (6/7) and 28.6 (2/7) (pregnant)	0 (0/7) (pregnant)
29 Asian^a^ 233 (2013-09-11) 233–2 (2014-02-12)	C (100, 16/16) (pregnant) vs. C (100, 16/16) (post-partum)	6.3 (1/16) (pregnant) vs. 12.5 (2/16) (post-partum) and 0 (0/16) (pregnant) vs. 0 (0/16) (post-partum)	0 (0/16) (pregnant) vs. 0 (0/16) (post-partum)
29/Asian 239 (2013-11-18)	B (100, 3/3) (pregnant)	33.3 (2/6) and 0 (0/3) (pregnant)	0 (0/6) (pregnant)
34/Asian 243 (2013-12-11)	C (100, 16/16) (pregnant)	12.5 (2/16) and 0 (0/16) (pregnant)	0 (0/16) (pregnant)
32/African 260 (2014-07-15)	D (100, 12/12) (pregnant)	100 (12/12) and 8.3 (1/12) (pregnant)	0 (0/12) (pregnant)
Summary of median % (range) S or P variants found during Pregnancy vs. Post-Partum		S gene variants	P gene variants
		10.4 (0–100) (pregnant) vs. 12.5 (0–100) (post-partum)	0 (0–9.1) (pregnant) vs. 0 (0–16.7) (post-partum)

^a^ Both pregnant and post-partum samples were analyzed from Cases 128, 160, 176, 196, 215, 223, 226 and 233 in the pre S/S and/or in the pre-C/C region (see Fig 1A and 1B). Case #176 was followed during 2 pregnancies, HBV pre-S/S/P sequences were analyzed in a sample collected after her first pregnancy (176–2 post-partum) and during her second pregnancy (176–3). HBV Pre-C/C sequences were analyzed at 3 time-points during first pregnancy (176), post-partum (176–2) and during second pregnancy (176–3) (see Figs 1 and 2).

^b^Mutations associated with immune escape include small S protein residues (i.e., 110, 120, 126, 129, 131, 133, 137, 139, 142, 145, 146, 147, 156, 166, 193, and 200); liver disease associated variants included deletions/insertions and/or start codon mutations in the pre-S1 and pre-S2 regions and the P110S mutation in the large S protein (see S1 Table).

^c^For Case ID#146 only the pre-S1 region was analyzed.

^d^Drug resistant (i.e., P) variants found included rtL80V (Case 160) and rtV191A (Case 217). The rtR153Q, rtQ182stop detected in Case 176 is associated with the classic VEM (i.e., G145R) variant in the overlapping surface (see S1 Table).

### Analysis of HBV Genome Evolution in Pregnancy and Post-Partum

**Fig 1:**
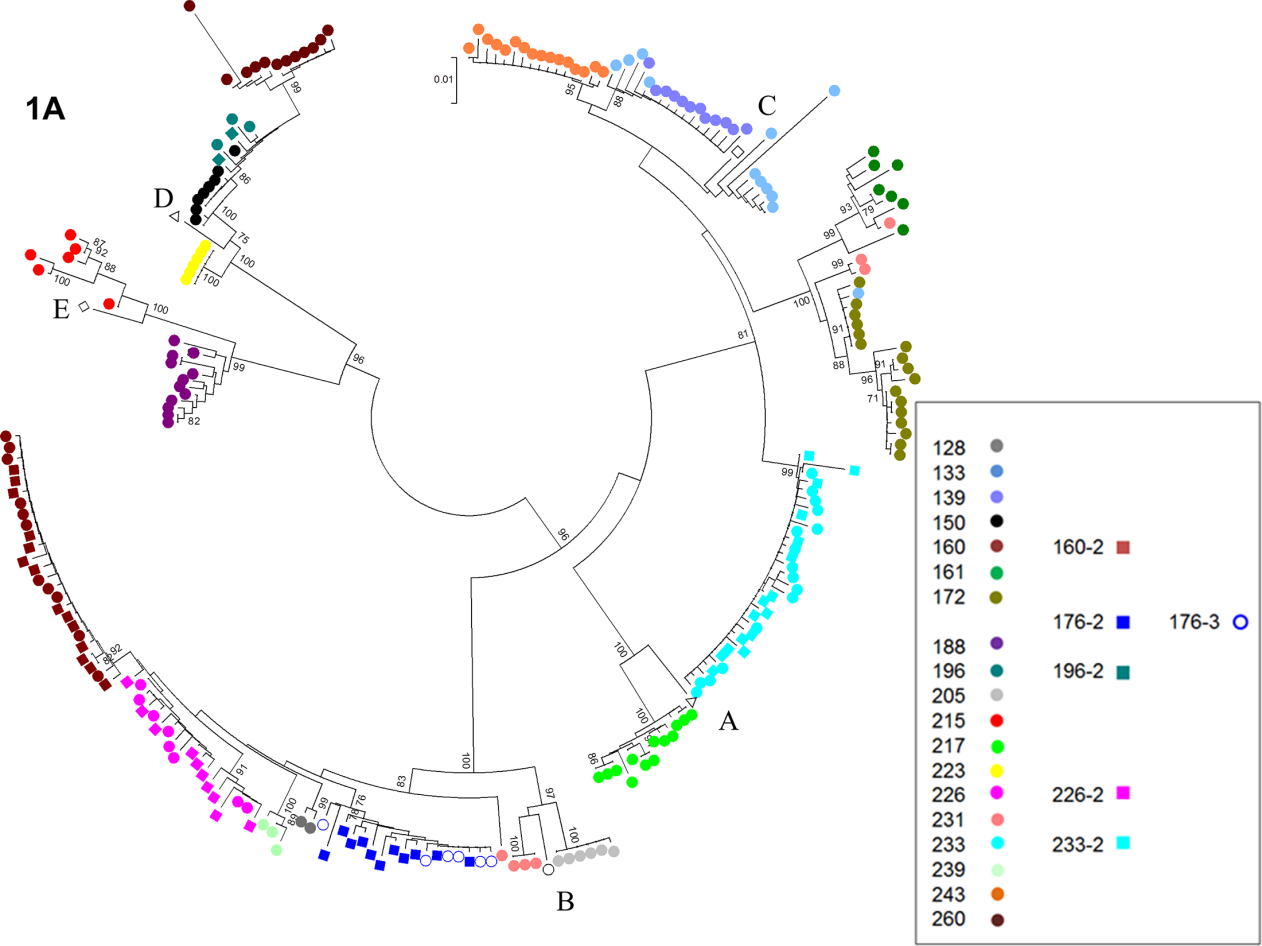
Neighbor-joining phylogenetic reconstruction of the HBV pre-S/S (A, N = 20), Pre-C/C (B, N = 11) and full genome (C, N = 3) using the bootstrap method. Clustering is prominent between individual cases, indicating a greater degree of variation between individuals, than amongst each of their viral quasispecies. Bootstrap values greater than 70 were considered significant. Case #146 was excluded from analysis as only the pre-S1 region was sequenced.

**Fig 2:**
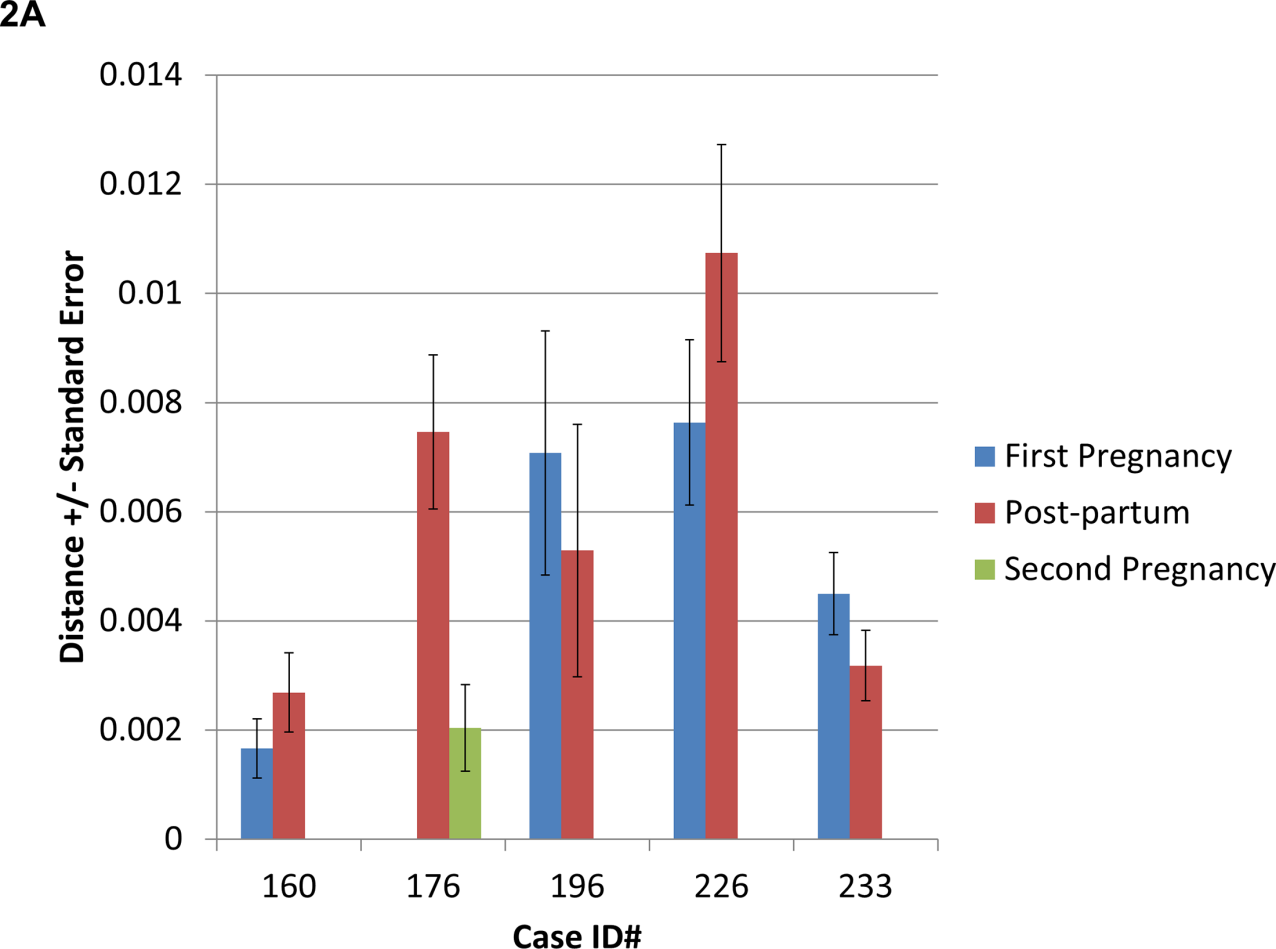
Comparison of distance amongst HBV quasispecies in patients during pregnancy and post-partum in pre-S/S (A, N = 5) and pre-C/C (B, N = 5) region. Measurement of distance within patient samples is shown and compared to measurement of distance within patient samples categorized by genotype. A comparison of relative evolutionary distances of patient samples collected at different time points is shown. Measurement of HBV distance within each individual sample is shown and compared to measurement of distance within individuals categorized by genotype. A comparison of relative evolutionary distances of HBV in each sample collected at different time points is shown.

Phylogenetic analysis revealed significant similarities in HBV pre-S/S/overlapping P in 20/21 cases (Case #146 was excluded as only the pre-S1 region was analyzed); pre-C/C (N = 11) and full genome sequences (N = 3) analyzed within pre- and/or post-partum time-points, which clustered together in a genotype and patient-specific fashion (Fig 1A, 1B and 1C). Comparison of pre-partum and post-partum HBV sequence divergence in pre-S/S and/or pre-C/C region amplified in 7 cases (ID# 160, 176, 196, 215, 223, 226, and 233), revealed no specific pattern of increasing or decreasing distance over time (Fig 2A and 2B). HBV sequences in 4 cases that experienced an ALT flare in antepartum and/or post-partum period (ID# 133, 150, 196–2, 239) showed evidence of positive selection (Table 3).

**Table 3:** Codon-based test of purifying selection averaging over all HBV sequence pairs in untreated or treated pregnant/ post-partum CHB carriers.

ID#	ALT pre/ post-partum, Antiviral Therapy	P Neutral	P Positive	P Purifying	Selection^d^
128	15 / 17, No	0.246	1.000	0.126	Neutral
133	10 / 24, No	0.075	0.037	1.000	Positive
139	23 / 19, No	0.591	0.296	1.000	Neutral
150	47 / 23, Yes	0.094	0.049	1.000	Positive
160; 160–2^a^	32 / 64, No	0.297; 0.927	0.150; 0.464	1.000; 1.000	Neutral; Neutral
161	26 / 47, No	0.249	0.119	1.000	Neutral
172^b^	16 / 64, No	0.140	1.000	0.067	Neutral
176–2; 176–3^a^ ^,^ ^b^ ^,^ ^c^	Unknown/18 /7, No	0.946; 0.508	1.000; 0.254	0.473; 1.000	Neutral; Neutral
188	34/16, No	0.204	0.105	1.000	Neutral
196; 196–2^a^ ^,^ ^b^	12 / 29, No	0.806; 0.022	1.000; 0.011	0.403; 1.000	Neutral; Positive
205	27 / 25, No	0.305	0.154	1.000	Neutral
215	43 / 41, No	0.367	0.183	1.000	Neutral
217	29 / 19, No	0.521	0.267	1.000	Neutral
223; 223–2 ^a^	20 / 56, Yes	1.000	1.000	1.000	Neutral
226; 226–2^a^	29 / 32, No	0.931; 0.900	1.000; 1.000	0.466; 0.450	Neutral; Neutral
231	32 / 13, No	0.374	0.188	1.000	Neutral
233; 233–2^a^ ^,^ ^b^	19 / 33, Yes	0.703; 0.643	0.350; 1.000	1.000; 0.322	Neutral; Neutral
239	6 / 26, Yes	0.031	0.014	1.000	Positive
243	15 / 16, Yes	0.223	1.000	0.104	Neutral
260	11/ 7, /No	0.292	1.000	0.143	Neutral

Notes:

^a^7 cases underwent HBV sequence analysis in both pregnancy and post-partum in pre-S/S and/or pre-C/C region (160, 176, 196, 215, 223, 226, 233).

^b^HBV sequence analysis in 4 cases (ID# 133, 150, 196–2 and 239) showed evidence of purifying selection. These cases also had ALT difference in pregnant compared to the post-partum period. *(P*-values less than 0.05 are significant).

^c^ Case #176 was followed during 2 pregnancies, HBV pre-S/S sequences were analyzed in a sample collected after her first pregnancy (176–2 post-partum) and during her second pregnancy (176–3). HBV. HBV Pre-C/C sequences were analyzed at 3 time-points during first pregnancy (176), post-partum (176–2) and during second pregnancy (176–3) (see Figs 1 and 2).

^d^dS and dN are the numbers of synonymous and nonsynonymous substitutions per site, respectively. The probability of rejecting the null hypothesis of strict-neutrality (dN = dS) in favor of the alternative hypothesis (dN < dS) and the test statistic (dS—dN) is shown. The variance of the difference was computed using the bootstrap method (1000 replicates). Analyses were conducted using the Nei-Gojobori method. The analysis involved 13 nucleotide sequences. All ambiguous positions were removed for each sequence pair and there were a total of 612 positions in the final dataset. Evolutionary analyses were conducted in MEGA V 6.0 (*Tamura K*, *Stecher G*, *Peterson D*, *Filipski A*, *Kumar S*. *MEGA6*: *Molecular Evolutionary Genetics Analysis version 6*.*0*. *Mol Biol Evol*. *2013; 30(12)*:*2725–9)*.

## Discussion

There is limited data on HBV evolution and variants in the context of pregnancy, and the effect of potent NA therapy, such as TDF. In the current study we examined HBV diversity as well as the presence of immune escape and drug resistant HBV mutants in 21 untreated and/or TDF-treated CHB patients during pregnancy compared to postpartum follow-up in 7/22 patients. Clonal sequence analysis of the HBV before and after parturition revealed that most cases analyzed expressed mutations in the Major Hydrophilic Region (MHR), from amino acid 120 to 160 of the small S protein, and the presence of minor S variants in the antigenic “a” determinant region. Mutations in the HBV “a” determinant region, as well as along entire S region, may affect antigenic conformation due to structural changes that alter antibody binding [16, 31–34]. The identification of such immune escape variants may be important due to the risk of HBV vertical transmission despite complete passive-active immunoprophylaxis [7, 8].

Maternal viral load and HBeAg status have been identified as risk factors for intrauterine transmission [11, 35]. Thus, current guidelines recommend consideration of NA therapy to reduce the risk of MTCT in highly viremic mothers (i.e., >6 log (10) IU/ml). There is limited data on HBV evolution in the context of NA-treatment during pregnancy. In one recent study of mothers treated with LMV or TDF during pregnancy, deep sequencing analysis showed increased frequency of LMV resistant variants post-partum in treated mothers [24]. Our previous clinical studies have observed that peri-partum HBV immune flares are common and TDF is safe and effective for use in pregnancy [23, 36]. In the current study, 28% (6/21) mothers had high level viremia warranting antiviral treatment to reduce vertical transmission risk, and 5 patients received Tenofovir treatment (1 declined treatment). Possible minor drug resistance mutations were identified at baseline in 3 cases (ID# 160, 176 and 217) (S1 Table). The selection test in pregnant and post-partum samples analyzed (Table 3) indicated that HBV sequences in most cases were under neutral selection, suggesting no net selective pressure, although in 4 cases with positive selection there was also evidence of immune active hepatitis B with increased ALT.

To date 68% (15/22) infants born to 21 CHB carrier mothers received the complete HBV immunoprophylaxis series and underwent testing confirming protective immunity. A significant proportion (31%, 7/22) of infants had missing data or did not undergo testing to confirm protective immunity. An entity called “occult HBV infection” has been described in up to 40% of infants despite completion of passive-active prophylaxis [37, 38]. Occult HBV infection is defined as persistence of low-level HBV DNA in serum, liver and lymphatic (i.e., immune cells) in individuals despite serum HBsAg negativity. This phenomenon has been linked to maternal HBeAg positivity and viremia as well as immunoprophylaxis and host immune selective pressure. Occult HBV infection is usually due to ongoing low-level HBV replication (i.e., according to the classic Taormina meeting definition) [39]. However, moderate-high replication without detectable HBsAg can also occur in the setting of S gene mutations producing a modified HBsAg (i.e., diagnostic escape mutant) that is not detected by commercial diagnostic detection assays [18]. In HBV endemic regions with widespread vaccination programs, mutation rate within the HBV S gene ‘a’ determinant along with occult HBV infection is increasing [40]. However as argued by others, an international consensus definition of VEMs needs to be established and will require the development of validated in vitro and in vivo assays for evaluating the infectivity, neutralizability, and clinical significance of particular variant/mutants identified [41]. Regardless, in the current study several cases carried minor S variants in pregnancy associated with classic vaccine escape (ID # 161, 176, 231, 233). Thus close follow-up of infants for possible breakthrough overt and/or occult hepatitis B is warranted.

We selected the HBV pre-S/overlapping P region for analysis, as it is the target of immune and drug selective pressure. Additionally the pre-C /C region is also a known target for host immune pressure, especially during HBeAg seroconversion flares. In 3 highly viremic cases, HBV full genome sequences were analyzed to confirm the predominant genotype and quasispecies evolution but this was not possible in all due to difficulties in HBV complete genome amplification in samples from patients with low viral load. We acknowledge that the sample size for our study is relatively small (In 21 mothers, with 22 infants; with pre-S /S sequence analysis (N = 20/21), and pre-C/C sequence analysis (N = 11/21), however this study provides further information and adds to the limited data in the literature regarding HBV evolution in pregnancy. It is recognized that that clonal sequencing approach only identifies HBV variants at 5–10% of the viral quasispecies. Next generation sequencing technology could provide a more in-depth view of the viral population and is an area for future investigation.

The current study is one of the few studies evaluating HBV evolution during pregnancy, especially under more potent NA therapy. We show that HBV viral diversity differs between pregnant patients, despite similar ethnic background (i.e., 65% of patients were Asian with genotype B or C). The HBV genome evolves during pregnancy and post-partum. In CHB mothers tested in pregnancy and post-partum, minor variants associated with vaccine escape, drug resistance and liver disease were identified, highlighting the need for ongoing maternal as well as infant follow-up. The data may help understand the natural history of chronic hepatitis B in pregnancy, especially which mothers would benefit from antivirals to reduce both overt and occult risk of hepatitis B vertical transmission.

## Supporting Information

S1 Table Summary of minor HBV variants in Pre-Surface/Surface and overlapping Polymerase region in 21 chronic hepatitis B carriers followed during pregnancy and/or post-partum. (DOCX)

## Abbreviations

ALT: alanine amino transferase
CHB: chronic hepatitis B
HBV: hepatitis B virus
HBsAg: HBV surface (S) antigen
HBeAg: HBV e antigen
C HBV: HBV core gene
P/RT: polymerase/reverse transcriptase gene
HCC: hepatocellular carcinoma
NA: nucleos/tide analog
HBIG: hepatitis B immune globin
LMV: Lamivudine
LSM: liver stiffness measurement
MTCT: mother-to-child transmission
MHR: Major Hydrophilic Region
TE: transient elastography
TDF: Tenofovir
VEM: vaccine escape mutant
