# Correction: Hepatitis B Virus (HBV) Variants in Untreated and Tenofovir Treated Chronic Hepatitis B (CHB) Patients During Pregnancy and Post-Partum Follow-up

**DOI:** 10.1371/journal.pone.0145898

**Published:** 2015-12-21

**Authors:** Boris Virine, Carla Osiowy, Shan Gao, Tong Wang, Eliana Castillo, Steven R. Martin, Samuel S. Lee, Kimberley Simmonds, Guido van Marle, Carla S. Coffin

Fig 1, “Neighbor-joining phylogenetic reconstruction of the HBV pre-S/S (A, N = 20), Pre-C/C (B, N = 11) and full genome (C, N = 3) using the bootstrap method,” and Fig 2, “Comparison of distance amongst HBV quasispecies in patients during pregnancy and post-partum in pre-S/S (A, N = 5) and pre-C/C (B, N = 5) region. Measurement of distance within patient samples is shown and compared to measurement of distance within patient samples categorized by genotype. A comparison of relative evolutionary distances of patient samples collected at different time points is shown,” do not appear in full. Please view the full Fig 1 and Fig 2 here.

**Fig 1:**
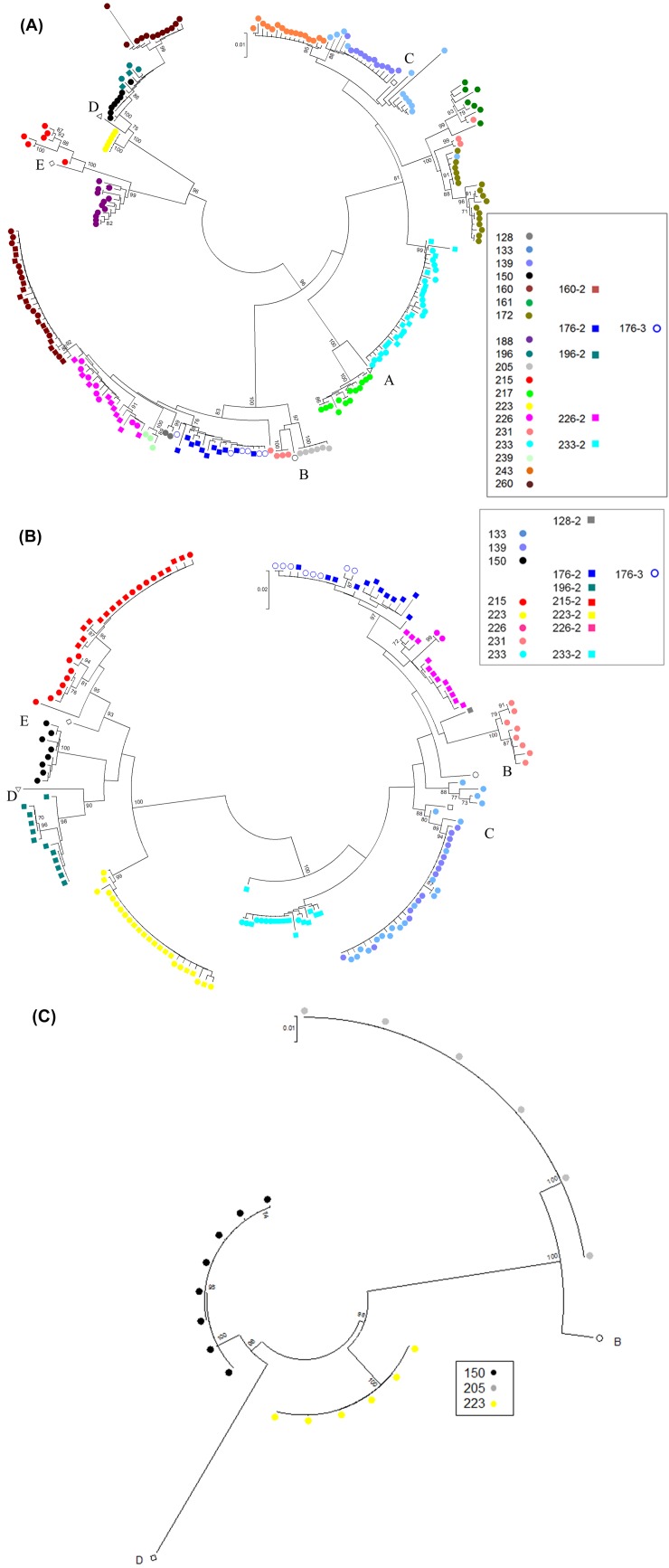
Neighbor-joining phylogenetic reconstruction of the HBV pre-S/S (A, N = 20), Pre-C/C (B, N = 11) and full genome (C, N = 3) using the bootstrap method. Clustering is prominent between individual cases, indicating a greater degree of variation between individuals, than amongst each of their viral quasispecies. Bootstrap values greater than 70 were considered significant. Case #146 was excluded from analysis as only the pre-S1 region was sequenced.

**Fig 2:**
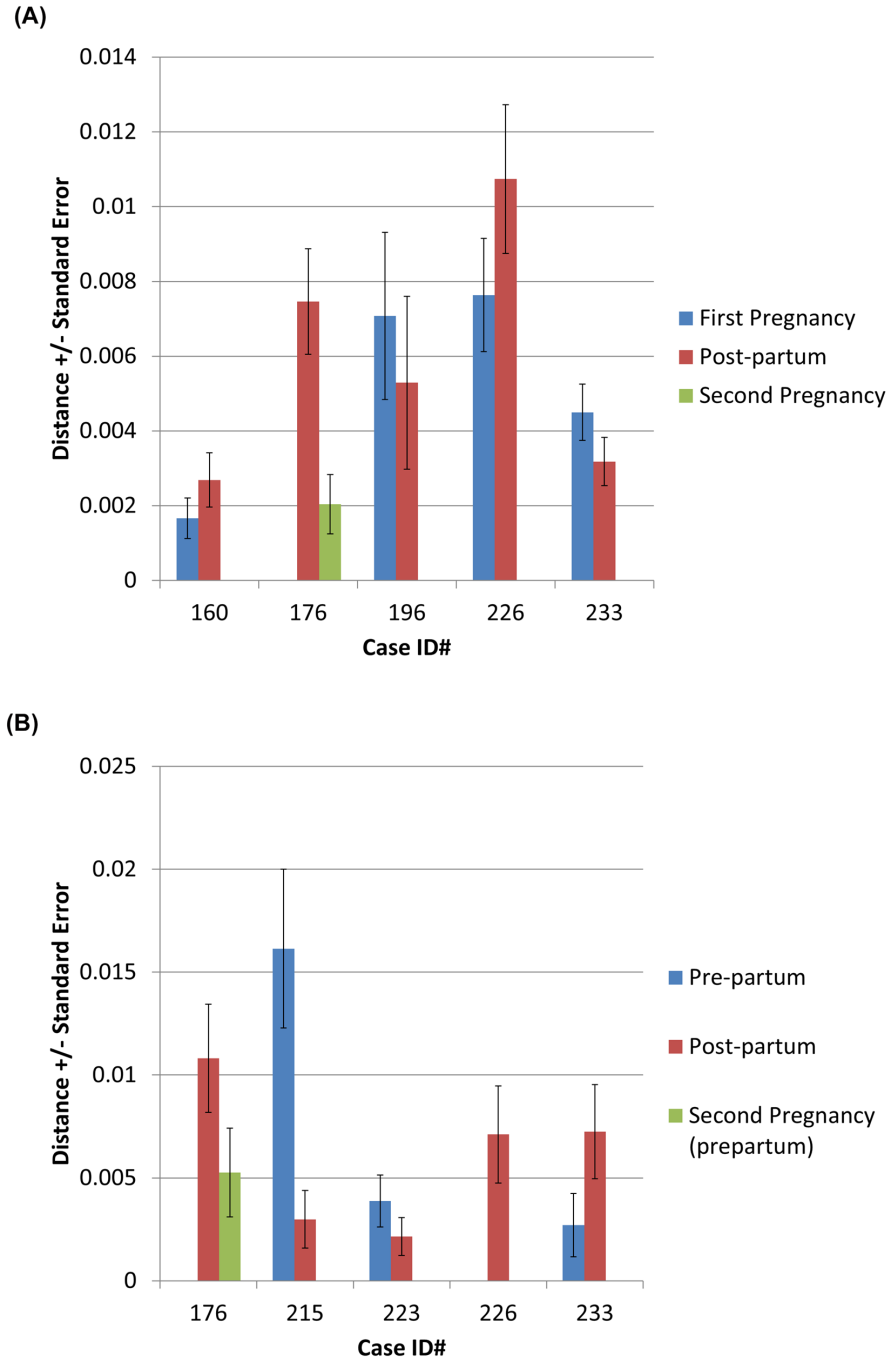
Comparison of distance amongst HBV quasispecies in patients during pregnancy and post-partum in pre-S/S (A, N = 5) and pre-C/C (B, N = 5) region. Measurement of distance within patient samples is shown and compared to measurement of distance within patient samples categorized by genotype. A comparison of relative evolutionary distances of patient samples collected at different time points is shown. Measurement of HBV distance within each individual sample is shown and compared to measurement of distance within individuals categorized by genotype. A comparison of relative evolutionary distances of HBV in each sample collected at different time points is shown.

## References

[1] Virine B, Osiowy C, Gao S, Wang T, Castillo E, Martin SR, et al (2015) Hepatitis B Virus (HBV) Variants in Untreated and Tenofovir Treated Chronic Hepatitis B (CHB) Patients during Pregnancy and Post-Partum Follow-Up. PLoS ONE 10(10): e0140070 doi:10.1371/journal.pone.0140070 26474400 10.1371/journal.pone.0140070 PMC4608582

