# Supplementary material for: Hepatitis B Virus (HBV) Variants in Untreated and Tenofovir Treated Chronic Hepatitis B (CHB) Patients during Pregnancy and Post-Partum Follow-Up
Source: PLoS One. 2015 Oct 16;10(10):e0140070. doi: 10.1371/journal.pone.0140070 (PMC4608582; doi:10.1371/journal.pone.0140070)
Supplement: S1 Table — (DOCX) [file pone.0140070.s001.docx]

**S1 Table: Summary of Minor Variants in Pre-S/S and overlapping P in 21 Patients followed during pregnancy and/or post-partum**

| ID | Pre-S/S position (%, N clones)  Pregnant vs. Postpartum | P/RT position (%, N clones)  Pregnant vs. Postpartum |
| --- | --- | --- |
| 128 | None detected | None detected |
| 133 | Premature stop codon aa 74 (42, 5/12),  PreS1 deletion aa 56 -117, (58, 7/12) | None detected |
| 139 | Premature stop codon aa 74 (8, 1/12) | None detected |
| 146 | None detected | None detected |
| 150 | sC147Y (12.5, 1/8) | None detected |
| 160;  160-2 ^a^ | None detected (1 indeterminant); None detected | rtL80V (9, 1/11); None detected |
| 161 | 1 indeterminant at aa 137, sP120L/sP120T (14, 1/7), sQ129H (28, 2/7), preS2 start codon mutation ATG🡪ACG (100, 7/7) | None detected |
| 172 | PreS2 start codon mutation ATG🡪ATA, (64, 11/17) | None detected |
| 176-2;  176-3^b^ | sT126A (91, 10/11), sC139Y (9,1/11), sG145R (9,1/11), sN146S (9, 1/11); sT126A (100, 6/6) | rtR153Q (9, 1/11); rtQ182stop (16, 1/6) |
| 188 | sQ129P (8,1/12), sC139S/s or C139Y (33, 4/12), sP142L (8, 1/12), sG145A (25, 3/12), PreS1 deletion aa 3-96, (100, 12/12) | None detected |
| 196;  196-2 ^a^ | None detected | None detected |
| 205 | None detected | None detected |
| 215 | PreS2 deletion aa16 - 22 (66, 4/6) or aa17-22 (33, 2/6) | None detected  T128I (6/7) |
| 217 | sT131D (15, 2/13), PreS1 start codon deletion (23, 3/13) | rtV191A (8,1/13) |
| 223 | None detected | None detected |
| 226;  226-2 ^a^ | sQ129R (10, 1/10), sM133T (10, 1/10)  PreS2 start codon mutation ATG🡪ATA (25, 2/8); PreS2 start codon mutation ATG🡪ATA (40, 4/10) | None detected |
| 231 | sP120S (42, 3/7), sT126N/sT126S (32, 3/7)  PreS1 premature stop codon (14, 1/7), PreS2 start codon mutation (ATG🡪ACG) (14, 1/7), PreS2 deletion (14, 1/7) vs. N/A | None detected |
| 233;  233-2 ^a^ | sC139R (6, 1/16); sC139R (6,1/16), sP142L (6, 1/16), sG145R (6, 1/16) | None detected |
| 239 | sT126A (16, 1/6), sT131A (16,1/6), sM133I (16, 1/6) | None detected |
| 243 | sT131A (6, 1/16), sC147Y (6, 1/16) | None detected |
| 260 | small S coding region 12-15 nucleotide deletion following aa138 (100, 12/12) | None detected |

^a^Seven cases were analyzed in both pregnancy and post-partum (160, 176, 196, 215, 226, 223, 233).

^b^ Case #176 was followed during 2 pregnancies, HBV pre-S/S sequences were analyzed in a sample collected after her first pregnancy (176-2 post-partum) and during her second pregnancy (176-3). HBV. HBV Pre-C/C sequences were analyzed at 3 time-points during first pregnancy (176), post-partum (176-2) and during second pregnancy (176-3) (see Figures 1-2).
